# Emerging trends in pharmacological treatment of inferior alveolar nerve sensory disorders: a scoping review

**DOI:** 10.4317/medoral.26519

**Published:** 2025-01-26

**Authors:** Bruno da Silva Mesquita, Ana Cláudia Amorim Gomes, Belmiro Cavalcanti do Egito Vasconcelos, Carlos Augusto Pereira Lago, Matheus Rodrigues dos Santos Arruda, Emanuel Sávio de Souza Andrade

**Affiliations:** 1 Department of Oral and Maxillofacial Surgery and Traumatology. University of Pernambuco, Recife, Pernambuco, Brazil; 2 Department of Dentistry. University of Paraíba, Araruna, Paraíba, Brazil

## Abstract

**Background:**

Sensory disorders of the inferior alveolar nerve, often arising from dental procedures, markedly impact the quality of life of patients. This article proposes a scoping review to analyze emerging trends in pharmacological treatment for these disorders, addressing scientific gaps and clinical practices.

**Material and Methods:**

The review followed the PRISMA-ScR protocol, conducting data searches across various databases, including PubMed and Cochrane, until March 2024. Case studies and series related to patients with sensory disorders of the inferior alveolar nerve undergoing pharmacological treatment were included.Parte inferior do formulário

**Results:**

Out of the initial 542 records, 7 studies were included after rigorous selection, displaying low bias risk. Medicinal therapies varied, encompassing analgesics, corticosteroids, anesthetics, and vitamin complexes. Dipyrone stood out in the preoperative phase, while prednisone proved effective in paresthesias. Selegiline, an MAO-B inhibitor, introduced an innovative approach.

**Conclusions:**

Agents such as selegiline offer an innovative approach to sensory restoration, while corticosteroids exhibit variable efficacy. Dipyrone is effective in preoperative pain control, and vitamin complexes provide regenerative and neuroprotective benefits. However, it is essential to consider that treatment success may vary and to address the individual needs of patients.

** Key words:**Sensory disorders, inferior alveolar nerve, pharmacological treatment, paresthesia, scoping systematic review.

## Introduction

Sensory disorders of the inferior alveolar nerve constitute a complex and challenging area in dental practice, encompassing a range of conditions from hypersensitivity to persistent neuropathic pain (1). Often resulting from dental surgical procedures, these disorders directly impacts patients quality of life, influencing not only their oral functions but also causing significant physical and emotional discomfort (2). In light of this scenario, it is imperative to explore innovative and effective therapeutic approaches for treating these conditions, emphasizing emerging trends in pharmacological treatment.

In the field of dentistry, particularly in oral and maxillofacial surgery, the search for pharmacological solutions that can provide relief to patients with sensory disorders of the inferior alveolar nerve is an ever-evolving area (3). Understanding new trends in this field becomes crucial for oral health professionals, paving the way for more effective and personalized approaches. However, the wide range of therapeutic options makes selecting the most appropriate approach a significant challenge in clinical practice.

Within this issue, there are not only gaps in scientific knowledge but also a lack of consensus on best practices in the pharmacological treatment of these disorders (4). The variety of causes and clinical manifestations of sensory disorders of the inferior alveolar nerve adds complexity to choosing the most appropriate treatment in specific cases. Making the construction of methodological frameworks difficult and resulting in studies with heterogeneous methodologies, this situation hinders the establishment of comparison parameters. This complexity favors the conduct of scoping reviews as high-evidence studies to support clinical practices. Furthermore, the rapid evolution of research in this area necessitates comprehensive and updated reviews that can synthesize the available scientific evidence, providing a solid foundation for clinical practice (5).

In this context, the present study proposes a scoping systematic review aimed at analyzing and synthesizing the existing scientific evidence on new trends in the pharmacological treatment of sensory disorders of the inferior alveolar nerve. Deepening the understanding of available therapeutic options, identifying research gaps, and proposing guidelines for future approaches are crucial objectives of this review. In doing so, the goal is not only to provide valuable information for oral health professionals but also to contribute to the ongoing advancement in the clinical management of these challenging conditions.

## Material and Methods

- Protocol and Registration:

This scoping review was structured based on the five-step methodology to provide a comprehensive and systematic overview of a specific research topic. Additionally, we followed the guidelines established by the 'PRISMA Extension for Scoping Reviews (PRISMA-ScR)', which offers a set of specific recommendations for the transparent and rigorous conduct of scoping reviews. These methodological approaches provide a solid framework for identifying, selecting, and critically analyzing the available literature on the subject at hand, thereby ensuring the reliability and validity of the review process (6,7,8).

- Identification of the Research Question

Research Focus: "What is the treatment trend for sensory disorders of the inferior alveolar nerve?". Open Science Framework Protocol Registration (OSF DOI 10.17605/OSF.IO/EM5BV). This review is registered in PROSPERO under the ID "CRD42023415605".

- Identification of Relevant Studies

Data search was conducted across various databases, including PubMed, Cochrane, Scopus, and Web of Science, up to March 07, 2024. Leveraging MeSH terms and precise keywords optimized the search process to retrieve studies concerning drug treatment of inferior alveolar nerve sensory disorders. This was independently performed by two authors (BSM and ACAG) using the following search strategy: (((“paresthesia” [MeSH Terms]) OR (“Mandibular Nerve Injuries” [MeSH Terms]) OR (“Mandibular Nerve Injuries”) OR (“paresthesia”) OR (“dysesthesia”) OR (“nerve injury”)) AND ((“inferior alveolar nerve”) OR (“trigeminal nerve” [MeSH Terms]) OR (“trigeminal nerve”)) AND ((“drug therapy”) OR (“Treatment”))). Additional records were manually identified through reference list searches of relevant publications. After removing duplicates using Mendeley Reference Manager, records were selected based on titles and/or abstracts for relevance. Full-text articles were then assessed for eligibility.

- Study Selection

The PCC (Population, Concept, and Context) strategy was employed for defining and selecting studies.

Participants (P): Patients diagnosed with inferior alveolar nerve sensory disorders.

Inclusion Criteria: Case reports, case series, and retrospective or prospective studies were considered for inclusion, involving participants of all age groups, genders, and ethnicities clinically or radiographically diagnosed with inferior alveolar nerve sensory disorders. These disorders included paresthesia, hypoesthesia, and other sensitivity alterations associated with this nerve, arising from various etiologies such as dental extractions, surgical procedures, trauma, or other related conditions, and who underwent pharmacological treatment. Various types of medications and therapeutic approaches were considered for analysis, including analgesics, anti-inflammatories, corticosteroids, and other relevant pharmacological interventions.

Exclusion Criteria: Applied to eliminate empirical and theoretical studies without a temporal limitation. Editorials, manuals, response letters, theoretical reflections, and publications that did not address the research question were also excluded.

Concept (C): Pharmacological treatment for inferior alveolar nerve sensory disorders.

The central concept of this scoping review was "pharmacological treatment for inferior alveolar nerve sensory disorders." It involved investigating pharmacological interventions used in the treatment of these disorders, focusing on effectiveness, safety, and trends in clinical practice. In this concept, the use of specific medications to alleviate symptoms associated with inferior alveolar nerve sensory disorders was highlighted. This included the use of analgesics for pain control, anti-inflammatories to reduce inflammation, corticosteroids to decrease the immune response, and other pharmacological therapies aimed at restoring or improving nerve sensitivity in this region.

Context (C): Pharmacological treatment for inferior alveolar nerve sensory disorders in evidence.

Pharmacological treatment for inferior alveolar nerve sensory disorders has been a topic of growing interest in dental research. Studies have investigated various medications and pharmacological therapies as potential treatment options, aiming to provide relief from symptoms and expedite the recovery of compromised nerve sensitivity.

- Data Charting

The full texts were meticulously examined in their entirety. When necessary, the authors were contacted to obtain additional information about the study design and clarification of data. Data from eligibility forms were recorded in Tables through independent double data entry performed by BSM and MRSA, with the validation process conducted by a third reviewer, ACAG.

- Data Clustering, Summary, and Presentation of Results

After analyzing the information from the studies, we compiled a narrative report of the findings, presenting descriptive data through basic numerical analysis that addresses the scope, nature, and distribution of the studies incorporated in the review. Individual and combined information was provided on the pharmacological therapies used in the treatment of sensory alterations of the inferior alveolar nerve, specifying the treatment response, resulting impacts, and the level of evidence of the studies discussed.

Extracted Data: Author, year of publication, number of participants, age, gender, type of diagnostic analysis, type of injury, implemented therapy, and follow-up time (Table 1); study outcomes (Table 2).

The descriptive information was organized based on the study design, and the included studies were categorized according to the level of evidence established by the Oxford Centre for Evidence-Based Medicine, with the risk of bias analyzed using the Cochrane Risk of Bias tool (RoB2.0).

Parte inferior do formulário

## Results

- Study Selection

**Figure 1:**
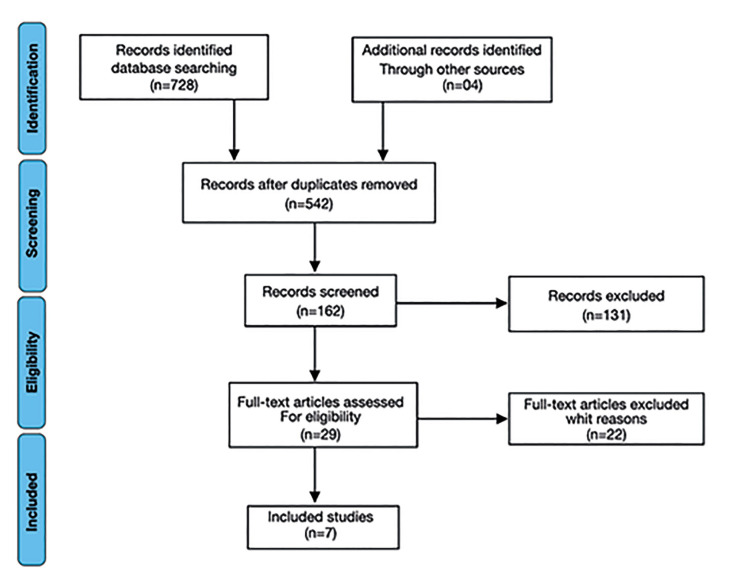
PRISMA flow diagram of the study selection process.

A total of 728 records were identified in the searched electronic databases: 637 in PubMed, 40 in Cochrane, 15 in Scopus, and 37 in Web of Science. In addition to these, 3 studies were included through additional manual searches. After removing duplicates, 542 records were selected for title and abstract screening. After this screening, 131 studies were excluded, leaving 29 for full-text reading. After applying the inclusion and exclusion criteria, 7 studies remained and were included in this scoping review (Fig. 1).

**Figure 2:**
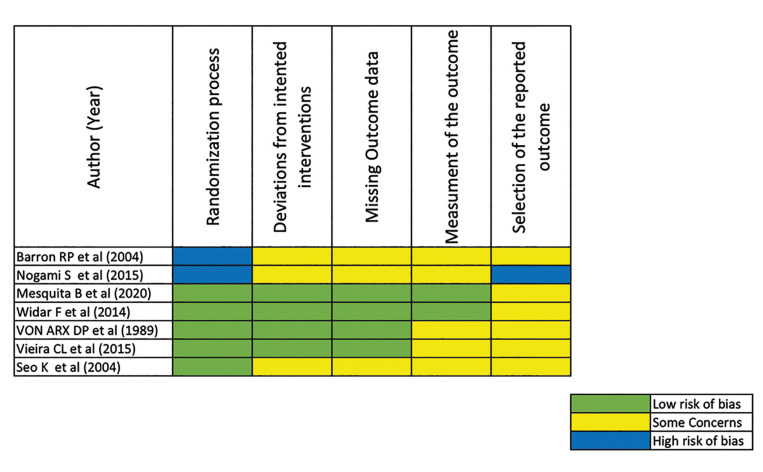
Risk of bias for the five domains included in the Cochrane Risk of Bias 2.0 tool for randomized trials (RoB2.0).

All Randomized Controlled Trials showed a "low risk of bias" for most of the assessed domains. However, two studies, Barron (2004) and Nogami (2015), presented a high risk of bias in the domain related to the randomization process, as they did not ensure the proper allocation of participants to groups (Fig. 2).

- Sample

A total of 702 individuals with sensory impairment of the inferior alveolar nerve were evaluated, with a mean age of 21.9 years. All studies focused on the pharmacological treatment of sensory alterations of the inferior alveolar nerve, including seven clinical trials and two case reports. These studies explored various treatments, including the use of analgesics, corticosteroids, anesthetics, vitamin B12 (mecobalamin), and medications targeting the central nervous system, such as selegiline and pregabalin.

**Figure 3:**
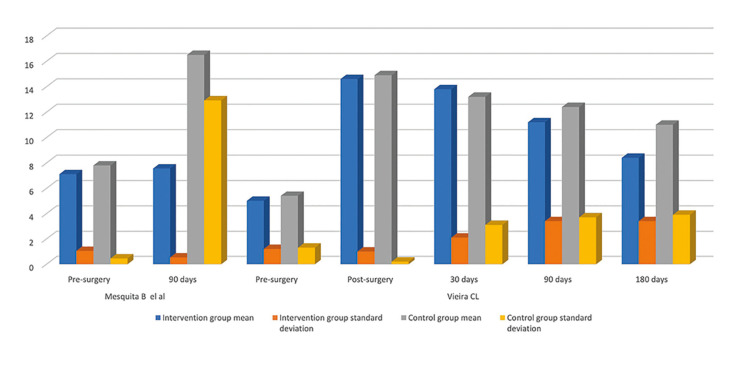
Column chart depicting comparative distribution between the intervention and control groups through the two-point static discrimination test.

The two-point static discrimination test was frequently employed to assess sensory recovery of the inferior alveolar nerve. Most studies showed an improvement in sensory discrimination over time, particularly in relation to the time elapsed since the injury. However, the improvement observed was not consistently associated with the pharmacological interventions. Data from these studies, including statistically significant results, are presented in Table 3 and Fig. 3.

Other evaluation methods were mentioned, including the visual analog scale, directional perception test, thermal test, touch sensitivity, electrical perception, and pressure test. In studies where there was a negative response to the proposed treatment, the visual analog scale was used for assessment. Dosage protocols and follow-up times varied.

## Discussion

A current trend in the treatment of peripheral nerve injuries involves the modulation of MAO-B through the administration of selegiline hydrochloride. The inhibitory action on this enzyme, which is released mainly in cases of direct nerve injuries and is responsible for the degradation of neurotransmitters such as dopamine, provides a restoration of sensory perception capacity. The development of this study on drug treatment for facial paresthesias related to the inferior alveolar nerve was based on scientific evidence available since 1989. Various studies were analyzed, including randomized trials, reviews, case reports, and the heterogeneity of the studies was a major challenge for the development of a logical and conclusive reasoning. However, we managed to identify the main causes, diagnostic methods, and treatments for this condition. Facial paresthesia is the most common type of dysfunction affecting the inferior alveolar nerve, a condition resulting in altered facial sensitivity and discomfort for patients. This condition typically stems from traumatic causes such as sagittal osteotomies in orthognathic surgeries, third molar extractions, or traumas related to endodontic procedures, where there is exposure, manipulation, or direct contact with the inferior alveolar nerve. In this context, the use of corticosteroids is well established, but the lack of standardization in the therapeutic protocol leads to random results (9-13).

Among the investigated corticosteroids, we identified two medications that did not show positive efficacy in the treatment of paresthesias related to the inferior alveolar nerve: betamethasone and dexamethasone (9,14). These corticosteroids are classified as high-potency, and this characteristic may be associated with the observed negative results. In contrast, prednisone, a corticosteroid of relatively low potency, demonstrated a positive response in the treatment of these paresthesias. This finding suggests that lower-potency corticosteroids, particularly prednisone, have the ability to directly modulate inflammation in the vasculonervous bundle, thus accelerating the functional recovery of the nerve. Still, in the realm of pharmacological treatments, it is worth highlighting the role of the analgesic dipyrone in the context of inferior alveolar nerve paresthesia. In contrast to high-potency corticosteroids, dipyrone proved effective in promoting the regeneration of the vasculonervous bundle, emphasizing the importance of specific pharmacological strategies in the effective management of this condition (15).

Dipyrone demonstrated efficacy in the preoperative period for the treatment of inferior alveolar nerve paresthesia, with a positive response attributed to several relevant factors: Dipyrone is known for its analgesic properties, playing a crucial role in pain relief (9,16). When paresthesia is associated with postoperative discomfort or pain, the administration of dipyrone can alleviate these symptoms, providing comfort to the patient. Additionally, dipyrone has anti-inflammatory properties that can modulate the inflammatory response triggered by direct or indirect traumas to the inferior alveolar nerve, especially during surgeries. This control of inflammation may contribute to the reduction of paresthesia incidence (17).

The ability of dipyrone to modulate sensitivity by blocking pain signal transmission is a relevant aspect when paresthesia is related to heightened or abnormal sensitivity. In this context, the influence of dipyrone can be beneficial. Furthermore, dipyrone exhibits antispasmodic properties, contributing to the relaxation of muscles in the region of the inferior alveolar nerve. This characteristic is particularly relevant when paresthesia is associated with excessive muscle contraction or spasms. Along the same lines, pregabalin has been studied as an option in this context. Pregabalin, an antiepileptic but also analgesic agent, has demonstrated efficacy in controlling neuropathic pain and reducing the abnormal sensation associated with paresthesia. Preliminary studies suggest that pregabalin may act on modulating neuronal excitability, providing relief of symptoms in patients with inferior alveolar nerve lesions. However, further relevant clinical research is needed to fully evaluate its efficacy, safety, and potential benefit as part of the therapeutic arsenal available for this specific condition, as only case reports have been identified as the type of study. The use of pregabalin should be carefully considered, taking into account factors such as dosage, treatment duration, and side effect profile, in order to offer patients an effective and well-tolerated treatment option (18,19). Although these drugs may play a crucial role in controlling pain and discomfort immediately before and after nerve trauma, it is important to highlight that other treatments have regenerative properties, such as vitamin complexes (20).

Vitamin complexes, although lacking robust evidence in the literature, are extensively used, standing out for providing promising results in the treatment of peripheral nerve injuries, including paresthesias linked to the inferior alveolar nerve. The integration of mecobalamin and the cytidine + hydroxocobalamin complex as therapeutic alternatives to address inferior alveolar nerve paresthesia offers a comprehensive view, balancing potential benefits and precautions. Mecobalamin, as an active variant of vitamin B12, has demonstrated beneficial properties in nerve regeneration and alleviation of neurological symptoms. Similarly, the cytidine + hydroxocobalamin complex, by combining essential nutrients with neuroprotective and antioxidant characteristics, provides comprehensive support to the nervous system, fostering conditions conducive to the recovery of the affected nerve (21,22).

In this perspective, it is crucial to highlight that, while vitamin complexes offer a preventive and regenerative approach, the literature also emphasizes alternative techniques, such as stellate ganglion block with lidocaine, as an intervention aimed at modulating the sympathetic nervous system. This technique, known to influence the head and neck region, including the area innervated by the inferior alveolar nerve, emerges as a possible tool to minimize the abnormal sensation associated with paresthesia, providing relief to patients. However, the need for additional studies to validate the effectiveness, safety, and clinical feasibility of this procedure is evident. Considerations regarding the duration of relief, potential side effects, and the careful selection of patients must be carefully examined to determine the practical applicability and benefits of this intervention in a broader clinical context (21,23).

In the pharmacological treatment of injuries associated with the inferior alveolar nerve, it is crucial to highlight some disadvantages and cautious considerations. The success of treatment may vary among patients, and not all may experience complete recovery. Additionally, possible side effects, drug interactions, and the individual response of each patient to the proposed treatments must be considered. Establishing realistic, individualized, and transparent expectations becomes essential in the therapeutic process. Clinical discussion, therefore, should address not only the potential benefits of these interventions but also the nuances and precautions associated. This balanced approach allows for a more comprehensive management of inferior alveolar nerve paresthesia, considering not only symptomatic treatment but also the responsible management of patient expectations and careful monitoring of the individual response to these interventions. In this context, the use of precise diagnostic methods is fundamental for choosing appropriate treatment (24).

The identification of various evaluation methods for inferior alveolar nerve paresthesia from different studies highlights the complexity and comprehensiveness required to understand this clinical condition. The Two-Point Static Discrimination Test, the Visual Analog Scale, the Directional Perception Test, the Thermal Test, Touch Sensitivity, Electrical Perception, and the Pressure Test emerge as distinct tools, each contributing a unique perspective on compromised nerve function. While the first offers a detailed analysis of tactile ability, the Visual Analog Scale provides a quantitative measure of paresthesia intensity. Thermal and directional perception tests explore the refined nuances of sensitivity. Touch sensitivity, electrical perception, and pressure testing complement the evaluation by considering different aspects of nerve function. The diversity of these methods, derived from various research methodologies, underscores the need for a multifaceted approach to encompass the various dimensions of inferior alveolar nerve paresthesia, facilitating the formulation of more comprehensive and personalized treatment strategies (25).

In addressing facial paresthesia related to inferior alveolar nerve disorders, in addition to the treatments discussed previously, various alternative therapies show potential efficacy. Among them are the use of platelet-rich fibrin (PRF), laser therapy, physiotherapy, massage, acupuncture, yoga, and aromatherapy. In certain circumstances, surgical intervention may be considered to address facial paresthesias, especially in cases of nerve compression or when severe traumatic injuries, such as nerve ruptures, are identified (26,27).

However, it is crucial to understand that therapies should not be viewed in isolation. The synergy between drug-based approaches and complementary therapies plays a crucial role in the effective management of paresthesia. The combination of these therapeutic modalities should be strategically applied, considering the specific needs of each patient. This integrated approach seeks to optimize therapeutic outcomes, promoting a more comprehensive and tailored response to the individual characteristics of the clinical presentation (28,29).

The present review faces several limitations that need to be considered when interpreting the results and recommendations. The heterogeneity of the analyzed studies, which include randomized trials, reviews, and case reports, makes it difficult to formulate consistent and generalizable conclusions. The lack of standardization in therapeutic protocols, especially regarding the use of corticosteroids, results in variability in outcomes, complicating the determination of optimal treatment. Additionally, many of the reviewed studies have small sample sizes or weak methodological design, which limits the robustness of the evidence. Direct comparison with other studies is further complicated by variations in study design, patient populations, and outcome measures, making it challenging to draw definitive conclusions across different research contexts. The absence of long-term studies and the scarcity of data on adverse effects and drug interactions also pose significant challenges. Finally, although some complementary and alternative therapies show potential, the current evidence is insufficient to fully validate their efficacy and safety, necessitating more rigorous clinical research.

## Conclusions

In summary, the treatment of inferior alveolar nerve paresthesia is complex and requires a multifaceted approach. Agents such as selegiline offer an innovative perspective on sensory perception restoration, while corticosteroids, despite being widely used, exhibit nuances in their efficacy. Dipyrone stands out in preoperative pain control, and the integrated approach of vitamin complexes provides regenerative and neuroprotective benefits. However, it is important to acknowledge that treatment success may vary, and carefully consider individual patient needs.

## Figures and Tables

**Table 1:** Literature data characterizing studies addressing inferior alveolar nerve sensory deficit treated with medications.

Author	Year	Study type	N		Type of Test	Type of injury
			Overall	intervention		
Barron RP, *et al.* (9)	2004	Randomized Controlled Trial	14	7	Electric Sensation	Traumatic
						Traumatic
Nogami S, *et al.* (21)	2015	Randomized Controlled Trial	47	21	Static Two-Point Discrimination	Traumatic
					Visual Analog Scale	Traumatic
Mesquita B, *et al.* (10)	2020	Randomized Controlled Trial	14	7	Static Two-Point Discrimination	Traumatic
					Directional perception	
					Touch Identification	
Widar F, *et al.* (11)	2014	Randomized Controlled Trial	37	25	Visual Analog Scale	Traumatic
Von Arx DP, *et al.* (14)	1989	Randomized Controlled Trial	550	280	Visual Analog Scale	Traumatic
Vieira CL, *et al.* (22)	2015	Randomized Controlled Trial	12	6	Static Two-Point Discrimination	Traumatic
					Nociceptive	
					Directional perception	
					Thermal sensitivity	
					Visual Analog Scale	
Seo K, *et al.* (12)	2004	Randomized Controlled Trial	26	20	Pressure Sensitivity	Traumatic
					Temperature Sensitivity	

**Table 2:** Literature data on outcomes of studies addressing inferior alveolar nerve sensory deficit treated with medications.

Author	Etiology	Type of therapy			Result	Follow -up
		Medication used	Daily dose	Duration		
Barron RP, *et al.* (9)	Extraction of third molar	Dipyrone	1g	Preoperative	Positive	8 days
		Dipyrone + Dexamethasone	1g + 8mg	Preoperative	Positive	
Nogami S, *et al.* (21)	Retromolar bone harvesting	Mecobalamin	1500 Ug	7 days	Positive	365 days
		Lidocaine	1%	Stellate ganglion block	Positive	365 days
Mesquita B, *et al.* (10)	Orthognathic surgery	Selegiline	5mg	30 days	Positive	90 days
Widar F, *et al.* (11)	Orthognathic surgery	Betamethasone	8mg	Preoperative	Negative	182 days
			4mg	Preoperative	Negative	
			4mg	1 day	Negative	
			16mg	Preoperative	Negative	
Von Arx DP, *et al.* (14)	Extraction of third molar	Dexamethasone	8mg	Preoperative	Negative	1h
Vieira CL, *et al.* (22)	Orthognathic surgery	Cytidine + hydroxocobalamin	5mg	60 days	Negative	182 days
Seo K, *et al.* (12)	Orthognathic surgery	Prednisolone	30mg	7 days	Positive	365 days
			15mg	4 days		
			5 mg	3 days		

**Table 3:** Objective data extracted from studies utilizing the two-point discrimination test as a diagnostic method.

Static two-point discrimination test (mm)						
Author	Follow up	Intervention group		Control group		p value
		mean	standard deviation	mean	standard deviation	
Mesquita B *et al.* (10)	Pre-surgery	7,11	1,05	7,8	0,45	0,24
	90 days	7,56	0,53	16,5	12,92	0,145
Vieira CL (22)	Pre-surgery	5,0	1,2	5,4	1,3	0,4
	Post-surgery	14,6	1,0	14,9	0,2	0,3
	30 days	13,8	2,1	13,2	3,1	0,5
	90 days	11,2	3,4	12,4	3,7	0,4
	180 days	8,4	3,4	11,0	3,9	0,1
